# Thrombosis-Associated Risk Factors in Pediatrics and Adults Treated with Asparaginase-Containing Chemotherapy for ALL: A Systematic Review and Meta-Analysis

**DOI:** 10.3390/curroncol33060368

**Published:** 2026-06-18

**Authors:** Jack T. Seki, Eshetu G. Atenafu, Hassan Sibai

**Affiliations:** 1 Leslie Dan Faculty of Pharmacy, University of Toronto, 144 College Street, Toronto, ON M5S 3M2, Canada; 2 Department of Biostatistics, University Health Network, 700 University Ave., Toronto, ON M5G 1X8, Canada; eshetu.atenafu@uhn.ca; 3 Division of Biostatistics, Dalla Lana School of Public Health, University of Toronto, 155 College Street, Toronto, ON M5T 3M7, Canada; 4 Division of Hematology, Department of Medical Oncology and Hematology, Princess Margaret Cancer Centre, University Health Network, 700 University Ave., Toronto, ON M5G 1X8, Canada

**Keywords:** thrombosis, asparaginase-containing chemotherapy, risk factors, adults and children

## Abstract

**Background**: Thromboembolism is a serious complication in acute lymphoblastic leukemia (ALL). This systematic review and meta-analysis evaluated thrombosis incidence and risk factors across populations receiving asparaginase-based therapy. **Methods**: From 214 studies (1994–2026), 58 met inclusion criteria, totaling 23,655 adult, pediatric, and mixed-population patients. Searches included Ovid MEDLINE, Embase, Cochrane CENTRAL, PubMed Central, and Google Scholar. Eligible studies were observational cohorts or clinical trials reporting thrombosis in ALL patients treated with asparaginase. Risk factors assessed included study design, asparaginase formulation, immunophenotype, gender, treatment phase, corticosteroid use, mediastinal mass, ABO blood group, body weight, and age. Random-effects models were used for meta-analysis, and risk of bias was assessed using ROBINS-I and RoB-2. **Results**: Adults had significantly higher thrombosis rates than children (*p* < 0.0001). Study design, asparaginase formulation, immunophenotype, and treatment phase differed significantly across age groups (*p* < 0.0001). T-cell ALL showed higher thrombosis rates than B-cell ALL (*p* < 0.0001). Significant pediatric risk factors included age ≥ 10 years, mediastinal mass, non-O blood type, and overweight/obesity (all *p* ≤ 0.0004). Gender and corticosteroid use were not significant predictors. Findings were limited by substantial heterogeneity across included studies. **Conclusions**: Thrombosis risk was multifactorial. Adults and older children had higher risk, and pediatric patients with overweight/obesity, mediastinal mass, or non-O blood type were particularly vulnerable. Thromboprophylaxis is advised for high-risk groups. This review was not registered and received no external funding.

## Simple Summary

Newer options like cal-asparaginase pegol are being used more often as the supplies of traditional asparaginase drugs continue to shrink. Thrombotic complications associated with asparaginase-containing chemotherapy have been increasingly recognized in both pediatric and adult ALL populations. Although the full picture is still developing, available evidence shows that clot prevention is important when patients have several risk factors. These risks appear to arise from a combination of factors, including the leukemia immunophenotype, patient age, treatment phase, and additional clinical characteristics identified in our analysis. Because asparaginase remains essential in ALL treatment, and these risks match what clinicians see in real practice, we strongly support using preventive measures like a heparin-derived blood thinner or antithrombin supplementation in patients who can safely receive them.

## 1. Introduction

Recipients of Pegylated (PEG)- and native *E. coli* asparaginase (NEA-ASP)- containing chemotherapy regimens for acute lymphoblastic leukemia (ALL) have experienced various toxicities, including thrombosis, in both adults and children [1,2]. Although thrombosis is a recognized complication of asparaginase-based therapy, its true incidence across different age groups remains uncertain. The reported risk factors have varied between adults and pediatric populations. The reported thrombosis incidence in children ranges from 1.7 to 36.7% [3], while significantly higher rates are observed in older patients (ages > 10 to <45.9) [4].

The wide variability likely reflects heterogeneity in study design, chemotherapy protocols, central venous catheter use, treatment duration, asparaginase formulations, dosing strategies, patient age, and thrombosis detection methods [5].

A meta-analysis of 17 pediatric studies reported a thrombosis rate of 5.2%, while adult cohorts treated with NEA-/Erwinase-ASP showed a slightly higher rate (5.9%, range 0–8.9%) [6,7]. A more recent large adult trial documented a substantially higher overall incidence rate of 16% in patients treated with NEA-ASP-containing intensive chemotherapy [8]. Mixed-age studies indicate significantly worse thrombosis outcomes in older children and young adults compared to children under 10 years of age [9,10,11]. In particular, two recent adult studies [12,13] reported even higher thrombosis rates (27.3% and 41%), in thromboprophylaxis-naïve patients. Although these studies had smaller sample sizes, the results strongly suggest the presence of inherent risk factors in older ALL patients. These risks may be related to pharmacological/non-pharmacological interventions, individual patient characteristics or a combination of both. Due to the lack of controlled risk variables in the standard arm, multiple pairwise comparisons using network meta-analysis were not carried out [14].

Although multiple studies have described thrombosis incidence within individual age groups or treatment regimens, no prior synthesis has simultaneously compared adults and children, evaluated formulation-specific risk (PEG-asparaginase vs. native *E. coli* vs. *Erwinia*), or integrated multivariable risk factors across immunophenotypes and treatment phases. Despite multiple prior studies having examined thrombosis in ALL, important gaps persist in understanding the following domains:

- How do thrombosis risks differ between adults and children receiving comparable asparaginase-based therapy?
- Do PEG-asparaginase and native/*Erwinia* formulations confer differential thrombosis risk when analyzed across age groups?
- Which patient-level factors (e.g., obesity, ABO blood group, immunophenotype) consistently predict thrombosis across studies?
- Are certain treatment phases associated with disproportionately higher risk?

In order to close these gaps, we conducted a comprehensive systematic review and meta-analysis to quantify thrombosis incidence and identify consistent risk factors among Philadelphia chromosome-negative ALL patients treated with PEG-asparaginase or native/*Erwinia* formulations.

The impetus for this research arose from our preliminary literature review, which revealed substantial variability in reported thrombosis rates and heterogeneous distribution of risk factors among adults and children treated with asparaginases NEA-/PEG-ASP. Although several studies evaluated thrombosis as either a primary endpoint or therapy-related toxicity, findings remained inconsistent despite the use of multivariable and Cox regression analyses. Thrombosis incidence may differ considerably in a meaningful way between adults and pediatric groups, notably in the context of interacting clinical and treatment-related factors. Therefore, our primary objective was to determine pooled thrombosis rates among ALL patients treated with PEG-/NEA-asparaginase-based regimens in the presence of associated risk factors across age groups. In doing so, we aimed to address the broader question of whether these findings may inform the potential role of thromboprophylaxis.

## 2. Materials and Methods

This study was designed as a systematic review and meta-analysis of observational studies and clinical trials evaluating thrombosis risk among pediatric and adult patients receiving asparaginase-containing chemotherapy for acute lymphoblastic leukemia (ALL). The study design was selected to enable pooled evaluation of thrombosis incidence and associated risk factors across heterogeneous patient populations and treatment protocols. The conduct and reporting of this systematic review and meta-analysis adhered to the PRISMA 2020 guidelines [15]. The completed PRISMA 2020 checklist is provided in Supplementary Table S1.

Heterogeneity statistics (I^2^) were calculated and reported only for pooled meta-analytic estimates presented in forest plots.

### 2.1. Eligibility Criteria

Eligible studies included randomized controlled trials, prospective or retrospective observational cohort studies, and case–control studies reporting thrombosis outcomes in pediatric, adolescent, young adult, or adult patients with Philadelphia chromosome-negative acute lymphoblastic leukemia (ALL) receiving asparaginase-containing chemotherapy. Studies were excluded if they involved non-ALL malignancies without separable ALL data, did not clearly report thrombosis outcomes, or administered routine pharmacologic thromboprophylaxis or erythropoiesis-stimulating agents (ESAs) to all patients. Trials containing both prophylaxis and non-prophylaxis arms were retained when extractable comparative data were available. Review articles, meta-analyses, pharmacoeconomic studies, case reports, and case series without statistical analysis were excluded. Abstract-only studies were included when sufficient outcome and risk-factor data were available.

### 2.2. Study Population and Treatment Characteristics

Included patients ranged from infancy through older adulthood and were treated with asparaginase-containing regimens in single- or multi-center settings. Accepted formulations included native *E. coli* asparaginase (NEA), PEG-asparaginase (PEG-ASP), and *Erwinia*-derived asparaginase when used for hypersensitivity. The primary outcome was venous thrombosis occurring after asparaginase exposure, including deep vein thrombosis (DVT), pulmonary embolism (PE), and cerebral venous sinus thrombosis (CVST). Where reported, thrombosis events were recorded as symptomatic or incidentally detected. Central venous catheter-associated thrombosis was included when not reported separately; where distinguishable, subgroup data were extracted. Mixed-age studies were categorized by reported age strata, and data were extracted separately when possible. Treatment phases analyzed included induction/re-induction, intensification, consolidation, and maintenance.

### 2.3. Study Selection and Duplicate Removal

**Figure 1:**
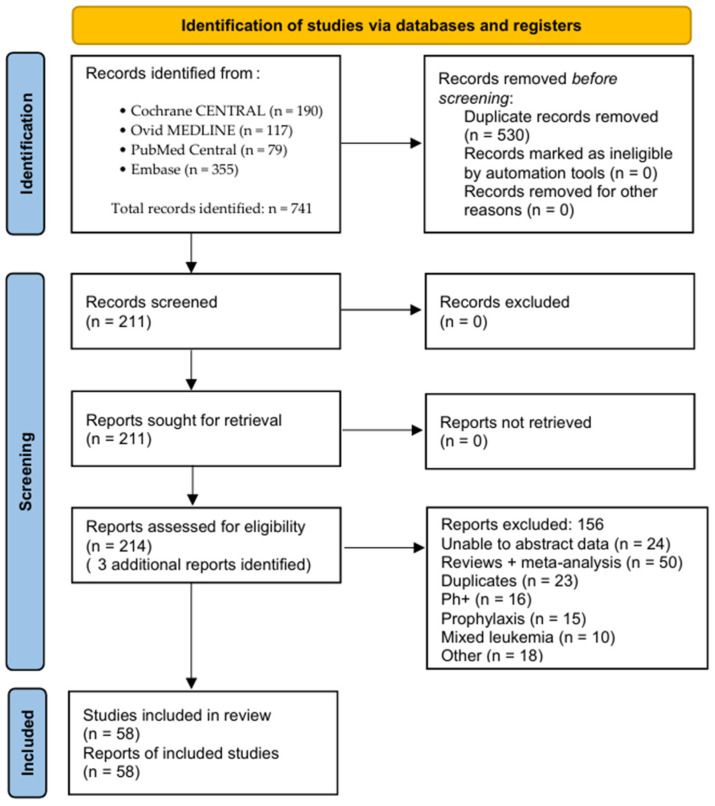
PRISMA 2020 flow diagram for new systematic reviews which included searches of databases.

Studies were identified through systematic database searches. Title and abstract screening were performed by the first author, with full-text review independently verified by the senior author; discrepancies were resolved by consensus. Duplicates were removed manually by comparing titles, authors, publication details, and unique identifiers across databases. Although dual independent screening at both stages is the preferred standard, this deviation is acknowledged as a methodological limitation. Data extraction used a standardized form capturing study characteristics, patient demographics, thrombosis incidence, treatment protocol, asparaginase formulation, treatment phase, and reported risk factors. We identified 214 studies for screening, of which 58 met inclusion criteria and were included in the meta-analysis (NEA = 27, PEG = 19, combined = 12), representing adult (*n* = 16), pediatric (*n* = 34), and mixed (*n* = 8) populations. The study-selection process is summarized in the PRISMA flow diagram (Figure 1).

### 2.4. Risk of Bias Assessment

Risk of bias (ROB) was assessed using the Cochrane Risk of Bias 2.0 tool for randomized trials and the 2020 ROBINS-I tool for non-randomized studies. Initial assessments were performed by the first author and independently reviewed by the senior author, with discrepancies resolved by consensus. ROB judgments informed qualitative interpretation of the evidence. This systematic review and meta-analysis study was not prospectively registered in PROSPERO, OSF, or INPLASY. However, a detailed internal protocol was developed prior to data abstraction, and eligibility criteria, data-extraction fields, and subgroup analyses were prespecified to minimize bias.

### 2.5. Search Strategy and Study Identification

Searches were conducted in PubMed Central, Ovid MEDLINE, Embase, Cochrane (Wiley), and Google Scholar for studies published between 15 June 1994 and 2 March 2026 (Supplementary Table S2a). Search terms included “thrombosis,” “thromboembolism,” “acute lymphoblastic leukemia,” “risk factors,” “pediatrics,” “adults,” “deep vein thrombosis,” and “pulmonary embolism.” The complete electronic search strategies for all databases, including full Boolean strings, limits, and the final search date, are provided in Supplementary Table S2b. Because Google Scholar does not support reproducible line-by-line Boolean strategies, we provide a narrative description consistent with PRISMA guidance. The search was last updated on 2 March 2026.

### 2.6. Data Handling and Identification of Risk Factors

Eligible studies were required to report thrombosis incidence in adult, pediatric, or mixed populations. Risk factors were abstracted when identified through multivariable analysis or Cox regression, supported by univariate analysis when applicable. Extracted variables included age, immunophenotype, corticosteroid exposure, ABO blood group, mediastinal mass, antithrombin III activity, inherited thrombophilia, body-weight category, treatment phase, asparaginase formulation, and central venous catheter use. Data entry was performed in structured spreadsheets and independently verified by the senior author and statistician.

### 2.7. Statistical Analysis

Categorical variables including VTE development, gender and other risk factors were presented as counts and percentages. An overall Chi-Square test was used to assess the VTE rates between pediatric and adult study data and across different doses of asparaginase. Forest plots were created to illustrate outcomes based on risk factors. The primary outcome of interest was the rate of VTE development. A Chi-Square association test was used to evaluate overall VTE rates across different doses of asparaginase. Logistic regression models were used to obtain odds ratio estimates of additional variables of interest while controlling the effect of pediatrics vs. adults. The unit of analysis was the study-level effect estimate. Effect estimates were extracted as odds ratios (ORs) with 95% confidence intervals (CIs) based on across-study aggregated counts. Adjusted estimates were preferentially used; otherwise, unadjusted ORs were calculated from available data. Substantial clinical and methodological heterogeneity was anticipated due to variability in study design, patient populations, thrombosis definitions, treatment phases, corticosteroid exposure, and asparaginase formulations. Therefore, all pooled analyses used random-effects models incorporating within-study error and between-study variability. Statistical heterogeneity was assessed using Cochran’s Q and quantified with the I^2^ statistic; τ^2^ was estimated under a random-effects model. Pooled estimates were interpreted as the mean of a distribution of true effects across heterogeneous contexts. Statistical significance was defined as *p* < 0.05. Prespecified subgroup analyses examined age group, treatment phase, asparaginase formulation, and study design. Forest plots were generated to display individual and pooled effect estimates. Adjusted ORs were obtained by fitting logistic regression models to aggregated event counts extracted from each study and do not represent pooled meta-analytic estimates. Heterogeneity statistics (I^2^) were calculated only for pooled meta-analytic ORs presented in forest plots, not for logistic regression models. All analyses followed established methodological guidance for observational data synthesis. These multivariable logistic regression models function as fixed-effect study-level models and do not incorporate between-study variance, in contrast to the random-effects structure used for pooled meta-analytic ORs.

In contrast, all non-MVA odds ratios presented in this review were calculated from across-study aggregated counts using random-effects meta-analytic models. For each pooled estimate, we report the corresponding 95% confidence interval and heterogeneity statistics (I^2^).

Sensitivity analyses were performed by repeating all primary meta-analyses after excluding studies judged at serious risk of bias according to ROBINS-I. Formal publication-bias testing (funnel plots, Egger’s regression) was not performed because several pooled analyses included fewer than 10 studies, making these methods statistically unreliable.

Statistical analyses were performed using version 9.4 of the SAS system for Windows (copyright ©2023 by SAS Institute, Inc., Cary, NC, USA) and the open-source statistical software R, version 4.5.2 (R Core Team [16], R Foundation for Statistical Computing, Vienna, Austria, 2025).

### 2.8. Study Inclusion and Dataset Finalization

**Figure 2:**
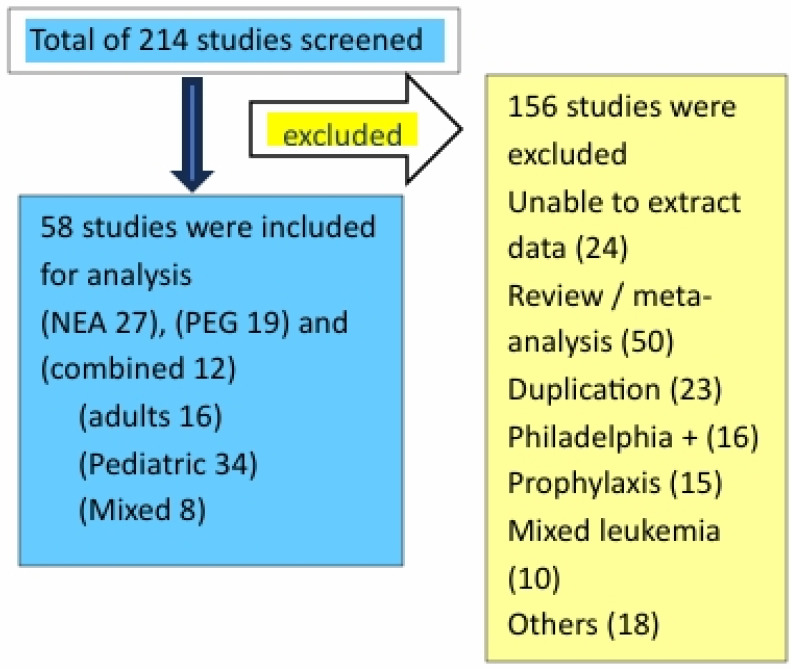
Studies used for the meta-analysis.

A total of 214 studies were screened, of which 58 met inclusion criteria for quantitative synthesis, comprising 23,655 patients. Studies were categorized into pediatric, adult, and mixed-age cohorts and further stratified by asparaginase formulation (Figure 2).

## 3. Results

Results are presented according to patient age group, asparaginase formulation, immunophenotype, treatment phase, and additional thrombosis-related clinical risk factors. Overall, adults consistently demonstrated higher thrombosis rates than pediatric patients across most study categories.

Significant thrombosis-associated risk factors identified in pediatric cohorts included older age, mediastinal mass, non-O blood group, and overweight/obesity status.

We summarize thrombosis incidences and associated risk factors in Table 1, comparing adults and pediatric populations. Overall, adults exhibited significantly higher thrombosis rates than pediatric patients (*p* < 0.0001, Table 2). The VTE rate was 12.66% in adult patients and 4.17% in pediatric patients (OR = 3.3352). Heterogeneity was substantial (I^2^ = 94.69%, Q(df = 48) = 903.9401, *p* < 0.0001).

Most included studies were observational and at moderate risk of bias, primarily due to confounding and incomplete outcome ascertainment. Only a minority of studies were judged to be low risk across all domains.

Adults in both single- and multi-center studies showed significantly higher risk of developing thrombosis than pediatric patients (*p* < 0.0001, Table 3). In single-center studies, adult patients recorded higher VTE rates (16.46%) than pediatric patients (7.12%). In multi-center studies, adult patients exhibited higher VTE rates (10.21%) compared to pediatric patients (3.44%). Multivariable analysis (MVA) confirmed these trends after adjusting single-center vs. multi-center studies (OR = 2.878, 95% CI 2.450–3.381, *p* < 0.0001) (Table 4).

Among PEG-ASP studies, adults had higher VTE rates (15.61%) compared to children (2.66%) [*p* < 0.0001]. Heterogeneity was high (I^2^ = 95.00%, Q(df = 43) = 860.3054, *p* < 0.0001). NEA-ASP studies showed adults exhibited a higher VTE rate (11.20%) than pediatric patients (6.28%); this difference was statistically significance (*p* < 0.0001). In BOTH-ASP studies, adult patients showed a higher VTE rate (8.19%) than pediatric patients (4.80%) [*p* = 0.0086] (Table 5). Multivariable analysis showed significantly higher VTE rates in adults than pediatric patients (*p* < 0.0001, OR = 3.041 (95% CI 2.589–3.572)), higher VTE rates in BOTH-ASP [*p* < 0.0001, OR = 1.359 (95% CI 1.153–1.601)], and those receiving NEA ASP compared to PEG-ASP recipients (*p* < 0.0001, OR = 1.771 (95% CI 1.524–2.058)) (Table 6).

**Figure 3:**
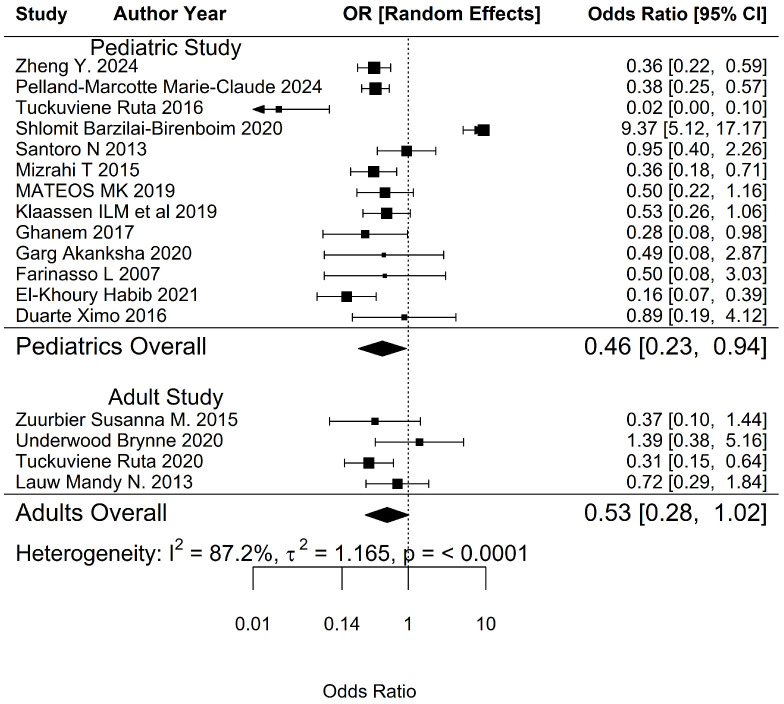
Forest plot comparing VTE risk in T-cell versus B-cell ALL. Pooled ORs were generated using a random-effects model. Studies included in this forest plot correspond to references [9,17,18,19,20,21,22,23,24,25,26,27,28,29,30,31,32]. Heterogeneity: I^2^ = 87.2%. Left-pointing arrows indicate that the confidence interval extends beyond the lower limit of the scale (0.01), while right-pointing arrows indicate that the confidence interval extends beyond the upper limit of the scale (10). Squares represent individual study estimates with their confidence intervals, and the diamond represents the pooled overall estimate.

Among B-cell studies, pediatric patients had a significantly higher VTE rate (5.53%) than adult patients (3.08%) [*p* < 0.0001]. In T-cell studies, pediatric patients had significantly higher VTE rates (12.15%) than adult patients (7.11%) [*p* = 0.0058] (Table 7). Multivariable analysis showed that pediatric patients had significantly higher thrombosis rates than adults [*p* < 0.0001, OR = 1.828 (95% CI 1.443–2.316)], and patients with T-cell had significantly higher VTE rates compared to B-cell patients [*p* < 0.0001, OR = 2.370 (95% CI 1.982–2.833)] (Table 8). Overall, B-cell patients posed a significantly lower thrombosis risk (*p* < 0.0001, OR = 2.333 (95% CI 1.981–2.747) compared to T-cell. Heterogeneity was considerable ((I^2^ = 87.23%, Q(df = 16) = 125.32, *p* < 0.0001), Figure 3, Table 9).

**Figure 4:**
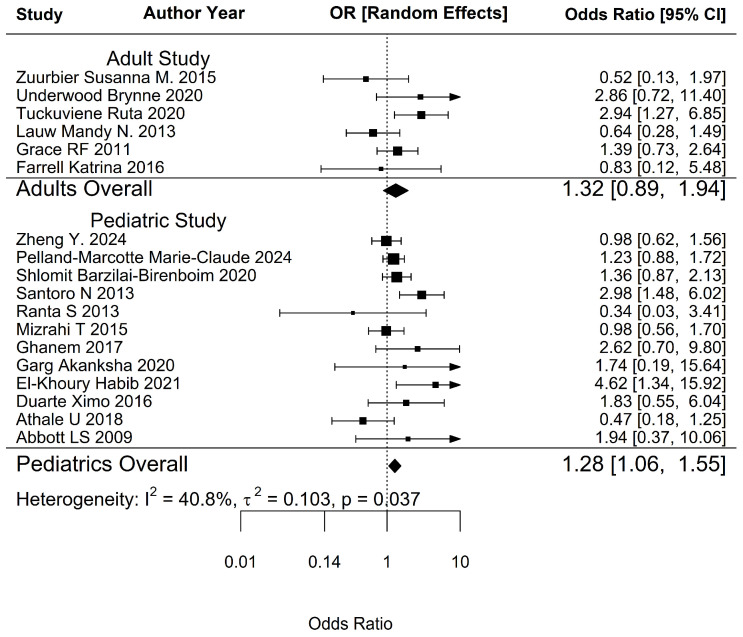
Forest plot comparing VTE risk in male versus female patients. Pooled ORs were generated using a random-effects model. Studies included in this forest plot correspond to references [9,10,17,18,20,21,22,23,24,26,27,28,30,31,33,34,35,36]. Heterogeneity: I^2^ = 40.8%. Adult patients undergoing induction/re-induction chemotherapy had significantly higher thrombosis risk (7.24%) compared to pediatric patients (2.27%) within the same treatment phases (*p* < 0.0001) (Table 11). However, no significant difference in thrombosis rate was found between treatment phases [(*p* = 0.0773), OR = 0.776, 95% CI 0.585–1.029] (Table 12).

No significant difference was found between adults and pediatric for both female and male gender cohorts in thrombosis risk [(*p* = 0.1719) and (*p* = 0.3973, respectively)] (Table 10). Among pediatric patients only, male patients had a significantly higher VTE rate OR = 1.28 (95% CI 1.06–1.55) (Figure 4). Heterogeneity was low-to-moderate (I^2^ = 40.80%, Q(df = 17) = 28.71, *p* = 0.0373).

Across subgroup analyses, heterogeneity ranged from low-to-moderate (gender: I^2^ = 40.8%) to substantial (adults vs. pediatrics: I^2^ = 94.6%). Sensitivity analyses excluding studies at serious risk of bias produced pooled effect estimates that were consistent in direction and similar in magnitude to the primary analyses across all major comparisons (adults vs. pediatrics, asparaginase formulation, immunophenotype, age group, and gender). Confidence intervals overlapped with those of the main analyses, and no changes in statistical significance were observed, indicating that the findings were robust to exclusion of serious risk of bias studies.

Among pediatric patients, no statistical difference was found between dexamethasone (Dex) recipients (2.43%) and prednisone (Pred) recipients (2.92%) [*p* = 0.2013, OR = 0.830, 95% CI 0.623–1.105] (Table 13).

Patients without a mediastinal mass had significantly lower thrombosis rates (6.04%) compared to those with a mediastinal mass (18.12%) (*p* < 0.0001) (Table 14).

Children with a non-O blood group type had a higher thrombosis risk compared to those with an O blood type (10.69% vs. 6.28%, *p* = 0.0004) (Table 15).

Overweight and obese (BMI 25 kg/m^2^–29.9/≥30 kg/m^2^ WHO) pediatric patients were at a significantly higher risk than non-overweight or underweight children (18.98% vs. 6.93%, *p* < 0.0001) (Table 16).

**Figure 5:**
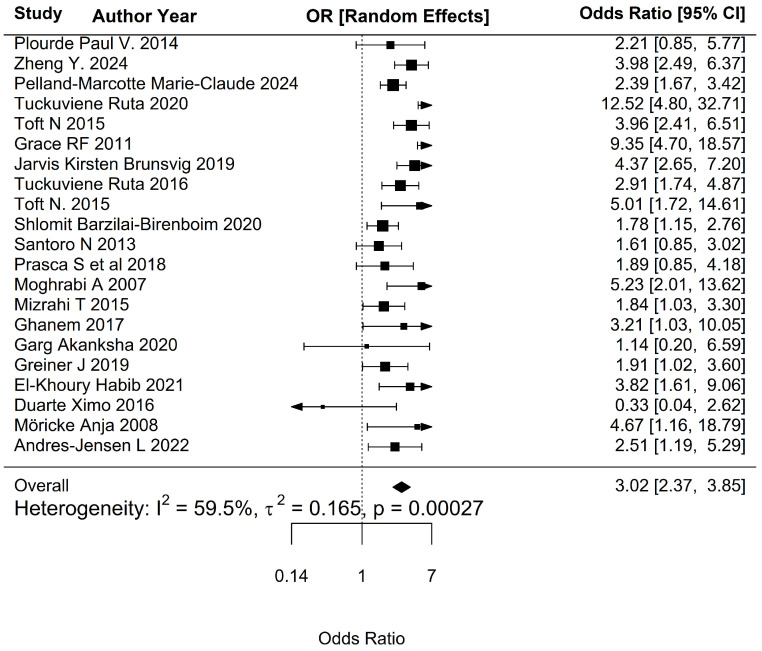
Forest plot comparing VTE risk in patients aged ≥10 years versus <10 years. Studies included in this forest plot correspond to references [9,10,11,17,18,20,21,24,26,27,28,30,32,37,38,39,40,41,42,43]. Pooled ORs were generated using a random-effects model. Heterogeneity: I^2^ = 59.5%.

Mixed-population patients (≥10 years, including adults and older children) had a similar thrombosis risk to pediatric patients (≥10 years) (9.85% vs. 9.57%, *p* = 0.7674, Table 17). At the same time, older children aged ≥10 years had an increased risk compared to those <10 years (*p* < 0.0001, HR = 3.02, 95% CI 2.37–3.85) (Figure 5). Heterogeneity was moderate (I^2^ = 59.48%, Q(df = 20) = 49.36, *p* = 0.0003).

**Figure 6:**
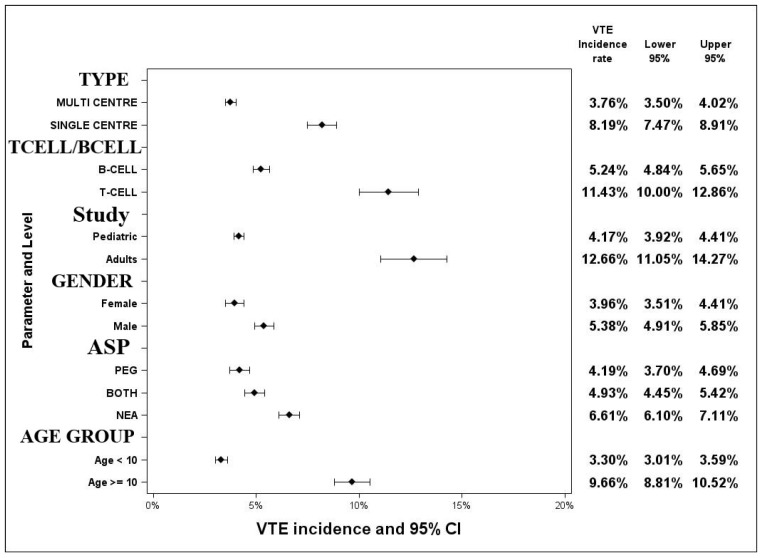
Forest plot. Illustrates higher VTE rates observed in single-center studies, T-cells, adults including patients age ≥ 10 y and patients treated with native *E. coli* asparaginase. Overall VTE incidence rates and 95% CI were generated using after combining summary count of each study together.

The forest plot (Figure 6) illustrates that higher VTE rates are observed in single-center studies, T-cell immunotype, recipients of NEA-asparaginase and patients with ages greater than 10 years.

## 4. Discussion

While the recently published protocol by Mkhwanazi et al. 2026 [44] focuses primarily on the overall prognostic impact of treatment-induced thrombosis in ALL, our study provides additional subgroup-level synthesis through age-stratified, formulation-specific, immunophenotype-specific risks, blood groups, weight-based predictive factors and phase-specific analyses. These methodological differences reduce redundancy and provide clinically actionable insights, thereby contributing complementary evidence. The result of this systematic review and meta-analysis found that the risk of thrombosis was prevalent and persistent throughout treatment history in adults and children undergoing ASP-containing therapy for ALL. To the best of our knowledge, thrombosis rates and potential risk factors in a direct comparison for adults versus children in ALL have not been thoroughly studied. The incidence rates varied by age: amongst children < 10 y of age (0.4% to 3.73%) and those ≥10 y, including adults (3.6% to 16.6% or higher) based on recently published multi-center studies [4,9,37].

We compared thrombosis risk factors across both age groups and found that adults (12.66%, *n* = 1643) had significantly higher thrombotic events than children (4.17%, *n* = 25,234) following exposure to ASP-containing chemotherapy (*p* < 0.0001) (Table 1), consistent with the results of earlier large mixed population studies and meta-analysis [4,6,9,37]. This difference remained significant across both single- and multi-center settings. However, these associations should be interpreted cautiously, as the included studies were predominantly observational, varied in methodological quality, and demonstrated substantial heterogeneity.

PEG-ASP has demonstrated greater and more sustained asparagine depletion than native *E. coli* asparaginase [45], but its thrombosis risk was initially unclear. Recent meta-analysis [46] and our data confirmed higher thrombosis rates with PEG-ASP, especially in adults (15.61% vs. 2.66% *p* < 0.0001) and in PEG-/NEA- combined protocols versus NEA alone (8.19% vs. 4.8%, *p* = 0.0086) (Table 1). Two large pediatric trials further supported PEG-ASP-associated thrombosis, especially among patients older than 10 years of age (*p* = 0.0001) [4,17]. In particular, older children (age > 10 y) and young adults (<18 years) more commonly exhibit higher thrombotic rates than younger pediatric patients, consistent with our findings [47] (Figure 5). These findings reflect associations rather than causal relationships, and residual confounding cannot be excluded.

Beyond age and asparaginase formulation, immunophenotype also appeared to influence thrombosis risk. We examined how immunophenotype influences thrombosis risk. In a combined analysis of pediatric and adult patients, the odds ratio favored lower VTE rates in B-cell patients (Figure 3 forest plot). However, in pediatric patients analyzed separately, both B-cell and T-cell immunophenotypes were associated with significantly higher thrombosis rates (Table 7). Multivariable analysis confirmed T-cell involvement as well as pediatric age were significant risk factors (*p* < 0.0001) (Table 8 and Table 9).

These findings align with a large retrospective pediatric ALL study (*n* = 2318) by Giordano P. et al. [48], which reported higher thrombosis rates in T-cell patients vs. non-T-cell patients (2.23% vs. 0.78%, *p* < 0.05) mostly during induction. Similarly, multivariable analyses have shown that T-cell immunophenotype, along with age < 1 year and ≥10 years, is independently associated with the development of thrombosis during the induction phase of treatment [18]. A recent meta-analysis by Zhang W. et al. [49] confirmed that T-cell phenotype in ALL is not only an independent risk factor but also contributes to a hypercoagulable state through excessive cytokine secretion. However, in a prospectively collected study, Athale UH et al. [50] reported that neither T-cell immunophenotype nor age ≥ 10 years remained significant risk factors for thrombosis in multivariable analysis. Given the variability in adjustment strategies and covariate reporting across studies, these immunophenotype-related associations should be interpreted as suggestive rather than definitive.

Separate analysis by gender shows no significant association with thrombosis risk among adults vs. pediatric patients (OR of 1.260, 95% CI 0.904–1.755; male *p* = 0.3973, female *p* = 0.1719) (Table 10). These findings are consistent with two large population-based cohort studies; first, a multivariable study (*n* = 2482) showed no gender difference within a year of ALL diagnosis (HR= 1.0, 95% CI 0.6–1.5, *p* = 0.9), though a Cox model suggested a non-significant trend for upper extremity thrombophlebitis in females (HR= 2.5, 95% CI 0.9–6.6, *p* = 0.07) [51]. A second study (*n* = 1820) found similar DVT risk across genders (HR1.01, 95% CI (0.68, 1.49), *p* = 0.9734) [52]. Similar findings were observed in elderly patients (age ≥ 65 years, *n* = 1088, *p* = 0.44) [53]. However, in adults and pediatric patients combined (mixed population), analysis showed that the male gender has higher thrombosis rates (Figure 4). These gender-related patterns should be interpreted cautiously due to heterogeneity in study design, population characteristics, and reporting practices.

Thrombosis is common during induction or re-induction, likely driven by the intensity of the treatment, and tumor lysis post-chemotherapy [6]. The thrombosis risk appears highest during induction therapy, particularly in hospitalized patients who often do not receive pharmacological prophylaxis. Prior studies have suggested that this setting may promote increased formation of neutrophil extracellular traps and elevated levels of cell-free DNA (c-f DNA), both of which have been associated with heightened thrombogenic potential [54]. However, these mechanistic pathways remain incompletely understood and should be interpreted as contributory hypotheses rather than definitive causal explanations. Thrombin generation differed significantly between patients with and without thrombosis (*p* < 0.005) [55]. Our data showed a significant difference between adults and children in thrombosis incidence during induction/re-induction (*p* < 0.0001) (Table 11). However, the event rates across treatment phases were similar (*p* = 0.0773) (Table 12).

Judicious use of glucocorticoids (dexamethasone and prednisone) is standard in ALL protocols to optimize clinical outcomes. Dexamethasone demonstrated superior CNS penetration and lymphoblast cytotoxicity (UK ALL 97/99 trial [56]), and lower thrombogenicity than prednisolone in two BFM protocols [57,58]. However, other protocols reported conflicting results or no difference in thrombosis risk [9,59]. Mechanisms underlying steroid- and ASP-related thrombosis have been well described [60,61,62,63]; however, corticosteroid dosing and bioequivalence remain unclear [64], suggesting pharmacologic difference may confound the dynamics of outcome. In our cohort, the corticosteroid toxicity rates in children (pred. 2.92% vs. dex. 2.43%, *p* = 0.2013) were comparable (Table 13).

Our study showed a significant association between increased thrombosis risk (*p* < 0.0001) and mediastinal mass (Table 14), consistent with findings from the NOPHO ALL 2008 trial (ages 1–45 years) [4], which reported a two-fold increase in thrombosis risk at diagnosis due to disease burden and vascular compression. Mediastinal mass and age > 10 years were major thrombosis risk factors during induction/consolidation therapy [19].

ASP-treated individuals showed age-related thrombosis risk, with older patients exhibiting higher thrombosis incidence linked to elevated procoagulant factors and pronounced hypofibrinolytic state [60] (age ≥ 10 y, OR 0.969 (0.788, 1.193), *p* = 0.7674) (Figure 5, Table 17). This trend was consistent with NEA-containing [37] and PEG-ASP-containing protocols [4], supported by ALL cancer registry data, showing elevated thrombosis risk in multiple adult age groups [53].

In a pediatric cohort of (*n* = 2123), non-O blood type was identified as an independent thrombosis risk factor, aligned with our findings (*p* = 0.0004, Table 15) [20]. Older age and non-O blood type were further validated as genetic predictors of thrombosis, particularly the rs2519093 genotype [21,33,65], although Jarvis KB et al. found no reproducible link between ABO single nucleotide polymorphism (SNP) and thrombosis in multiple Cox regression analysis [38].

Retrospective data from 1443 pediatric patients [66] showed that overweight/obese patients (BMI 25 kg/m^2^) had increased treatment-related toxicities including significantly higher thrombosis risk. Younger underweight patients also showed elevated thrombosis risk. Similarly, a separate cohort study (*n* = 294, age 1–21 years) confirmed obesity as an independent risk factor, with the majority of events occurring during induction post PEG-ASP in patients with CVCs [39]. Our analysis correspondingly found that overweight/obese (BMI 25 kg/m^2^–29.9/≥30 kg/m^2^ WHO) pediatric patients had a significantly higher thrombosis risk than normal/underweight patients (*p* < 0.0001, OR = 3.147 [95% CI 2.518–3.934]) (Table 16). These clinical and biological factors highlight the multifactorial nature of thrombosis risk in ALL.

Insights into thrombosis risk and potential prevention strategies.

Our data, supported by the existing literature, indicate that thrombosis risk in ALL is multifactorial, extending beyond leukemia-related procoagulants. Rodriguez et al. described multiple overlapping risk factors, especially during induction, as key thrombosis contributors [67]. Elevated risk has been linked to the male gender, asparaginase use, steroids and CVC placement [45,68,69].

Although our analysis provided insights into dynamic interaction between patient characteristics and thrombosis risk factors, the precise combination of constitutive risk factors underlying thrombosis susceptibility remains unclear. The identification of high-risk profiles may help to guide closer clinical monitoring in vulnerable patients [45,67]. However, the current evidence base—largely observational and heterogeneous—does not support broad recommendations for routine thromboprophylaxis. Preventive strategies, when considered, should be individualized and ideally evaluated within prospective, protocol-specific studies. Importantly, thrombotic events in children, including cerebral sinus thrombosis, can be safely managed pharmacologically without unnecessary delays or discontinuation of treatment, as premature truncation is associated with poorer outcomes [12,13,45].

Asparaginase-containing treatment regiments and their clinical implications.

A recent adult study reported thrombosis as the most frequent adverse events (35.6%) with PEG-ASP, showing a trend toward higher rates than NEA-ASP (*p* = 0.061), though direct comparisons remain inconclusive [70]. Our analysis, however, strongly indicates higher thrombosis risk in adults than children while receiving PEG-ASP (*p* < 0.0001) and in protocols where both PEG-/NEA-ASP are used in combination (*p* = 0.0086) (Table 1).

Older age may impair patient tolerance to intensive asparaginase-containing therapy. Patients who tolerated intensive asparaginase treatment had significantly better event-free survival (EFS); however, children ≥ 9 years were less likely to complete treatment [71,72]. Thrombosis is a leading cause of treatment discontinuation [73]; in a French study, 50% of NEA-ASP recipients stopped therapy after thrombotic events. Reduced asparaginase exposure was associated with lower median survival (19 vs. 53 months, *p* = 0.06) and reduced disease-free survival (14 vs. 58 months, *p* = 0.05), underscoring the relapse risk from premature discontinuation [74]. These associations should be interpreted cautiously, as study-level variability and unmeasured confounding may influence the observed patterns. A detailed appraisal of methodological limitations follows below.

### Limitations

It is important to emphasize that the majority of included studies were observational in design, and several were judged to have moderate or serious risk of bias. As such, the associations identified in this meta-analysis should not be interpreted as causal. Observational data are inherently vulnerable to residual confounding, unmeasured covariates, and selection bias.

Thrombosis in ALL appears to result from a complex interplay of established and potentially unrecognized risk factors that may act additively or synergistically to increase thrombotic risk [3]. The complex interplay of risk factors likely contributes to variability across studies and explains why our findings align with some studies while differing from others. A major limitation of this meta-analysis was the substantial heterogeneity among included studies. Differences in selection criteria, study quality, reporting of patient characteristics, age definition (e.g., defining adulthood at ≥15 years), and limited available data may have affected the interpretation and generalizability of the findings. Because the included studies were predominantly observational and varied in quality, causality cannot be established. Substantial clinical and methodological heterogeneity was present across studies, including differences in thrombosis definitions, treatment protocols, age distributions, and reporting practices, which limits the precision and generalizability of pooled estimates. Together, these sources of variability, along with the potential for publication bias and limited availability of fully reported data, may affect the interpretation and generalizability of our findings (Table S2a) [4,8,9,11,19,22,23,24,33,39,61,71,75,76,77]. First, because arterial thrombosis is rare, its inclusion within undifferentiated “thrombosis” categories may have introduced outcome misclassification and modestly biased our pooled venous thrombosis estimates. This limitation highlights the need for more consistent, site-specific reporting in future studies.

Second, initial title and abstract screening was performed by a single reviewer, although full-text eligibility was independently verified by a second reviewer.

Third, studies in which all patients received thromboprophylaxis or erythropoiesis-stimulating agents (ESAs) were excluded because prophylaxis modifies baseline thrombosis risk; however, trials that included both prophylaxis and control arms were retained. This may limit generalizability to settings in which prophylaxis is routinely applied.

Fourth, *Erwinia*-based asparaginase is used in cases of hypersensitivity to other formulations. In a large compassionate-use trial [40], thrombosis rates with *Erwinia* asparaginase were similar to or lower than those observed with comparator products. Inclusion of such data could influence our thrombosis estimates and represents a potential limitation.

Fifth, because the number of eligible studies was small, we did not perform formal sensitivity analyses excluding studies at high risk of bias. Additionally, risk-of-bias assessments were conducted by a single reviewer and verified by a senior author, which may introduce some degree of subjectivity despite the use of structured tools.

Finally, despite applying strict inclusion and exclusion criteria—specifically excluding studies involving Philadelphia chromosome-positive patients, routine prophylactic anticoagulation, or arterial thrombotic events—variability across published cohorts means that some included studies reported limited instances of these features. These occurrences were infrequent, did not represent the primary study populations, and reflect the unavoidable heterogeneity of real-world clinical datasets. These factors were considered during data extraction to minimize their impact on the overall findings.

## 5. Conclusions

This systematic review and meta-analysis demonstrated significantly increased thrombosis risks among adults and older children (≥10 years) with ALL receiving PEG-asparaginase-containing chemotherapy particularly during the induction and re-induction phases. The observed thrombosis profile appeared multifactorial, with associations identified across immunophenotype, obesity, patient age, treatment phase, mediastinal mass and ABO blood group. In pediatric and mixed cohorts, the B-cell immunophenotype and T-cell immunophenotype were both associated with increased thrombosis risk, while overweight/obesity and mediastinal mass further amplified susceptibility. These findings may assist in identifying high-risk patient groups for closer monitoring and individualized thromboprophylaxis strategies. However, risk stratification alone does not justify routine thromboprophylaxis; randomized evidence and protocol-specific guidance should drive practice. Ultimately, the evidence synthesized here underscores patterns of association rather than causation; only rigorously designed prospective studies will determine whether targeted thromboprophylaxis can meaningfully and safely reduce VTE risk in ALL.

## Figures and Tables

**Table 1:** Potential risk factors, VTE rate comparison between adults and pediatric groups, and statistical findings.

Risk Factor Categories	Adult Studies (*N*)	VTE *n* (%)	Pediatrics Studies (*N*)	VTE *n* (%)	Chi-Square *p*-Value
Thrombosis rates	1643	208 (12.66%)	25,234	1051 (4.17%)	<0.0001
Single-center	644	106 (16.46%)	4960	353 (7.12%)	<0.0001
Multi-center	999	102 (10.21%)	20,274	698 (3.44%)	<0.0001
PEG studies	743	116 (15.61%)	5531	147 (2.66%)	<0.0001
NEA studies	607	68 (11.20%)	8613	541 (6.28%)	0.0001
Both	293	24 (8.19%)	7370	354 (4.80%)	0.0086
B-cell	1783	55 (3.08%)	8198	453 (5.53%)	<0.0001
T-cell	380	27 (7.11%)	1276	155 (12.15%)	0.0058
Female	1195	45 (3.77%)	4002	188 (4.70%)	0.1719
Male	1532	86 (5.61%)	5160	320 (6.20%)	0.3973
Ind/Re-Ind	525	38 (7.24%)	12,689	288 (2.27%)	<0.0001
Intens/Cons/M	0	0	372	16 (4.30%)	NA
Dexamethasone	NA	NA	3247	79 (2.43%)	0.2013 *
Prednisone	NA	NA	4181	122 (2.92%)	
Mediastinal mass (no)	NA	NA	3213	194 (6.04%)	<0.0001 *
Mediastinal mass (yes)	NA	NA	287	52 (18.12%)	
Non-O blood group	NA	NA	1216	130 (10.69%)	0.0004 *
O blood group	NA	NA	907	57 (6.28%)	
Normal/under_wt	NA	NA	4791	332 (6.93%)	<0.0001 *
Overwt/Obese	NA	NA	669	127 (18.98%)	

Footnote: *N* = total sample size. Ind/Re-Ind = induction/re-induction. Intens/Cons/M= induction/intensification/maintenance. NA = data not available. *—*p*-values based on levels determined from pediatrics data only.

**Table 2:** Adults vs. pediatrics on VTE.

	Study		
	Adults (*N* = 1643)	Pediatric (*N* = 25,234)	*p*-Value
Outcome, *n* (%)			<0.0001 ^1^
Non-VTE	1435 (87.34%)	24,183 (95.83%)	
VTE	208 (12.66%)	1051 (4.17%)	

^1^ Chi-Square *p*-value; OR = 3.3352 (95% CI 2.8478–3.9060).

**Table 3:** Association between single-center/multi-center studies and adults vs. pediatric.

Pediatrics on VTE		Study		
Type		Adults	Pediatric	*p*-Value
Single-Center		(*N* = 644)	(*N* = 4960)	
	Outcome, *n* (%)			<0.0001 ^1^
	Non-VTE	538 (83.54%)	4607 (92.88%)	
	VTE	106 (16.46%)	353 (7.12%)	
Multi-Center		(*N* = 999)	(*N* = 20,274)	
	Outcome, *n* (%)			<0.0001 ^1^
	Non-VTE	897 (89.79%)	19,576 (96.56%)	
	VTE	102 (10.21%)	698 (3.44%)	

^1^ Chi-Square *p*-value; OR = 2.571 (95% CI 2.033–3.252) for single- and 3.189 (95% CI 2.564–3.967) for multi-center.

**Table 4:** Multivariable analysis (MVA).

Effect	*p*-Value	OR (95% CI)
Study (Adults vs. Pediatric)	<0.0001	2.878 (2.450, 3.381)
Type (Single-Center vs. Multi-Center)	<0.0001	2.072 (1.836, 2.338)

Footnote: MVA = multivariable analysis effect estimate reported directly by the original study authors. Adjusted ORs shown were obtained from author-generated logistic regression models using aggregated event counts.

**Table 5:** Association between PEG/NEA studies and adults vs. pediatrics on VTE.

		Study		
ASP		Adults	Pediatric	*p*-Value
PEG		(*N* = 743)	(*N* = 5531)	
	Outcome, *n* (%)			<0.0001 ^1^
	Non-VTE	627 (84.39%)	5384 (97.34%)	
	VTE	116 (15.61%)	147 (2.66%)	
NEA		(*N* = 607)	(*N* = 8613)	
	Outcome, *n* (%)			<0.0001 ^1^
	Non-VTE	539 (88.80%)	8072 (93.72%)	
	VTE	68 (11.20%)	541 (6.28%)	
BOTH		(*N* = 293)	(*N* = 7370)	
	Outcome, *n* (%)			0.0086 ^1^
	Non-VTE	269 (91.81%)	7016 (95.20%)	
	VTE	24 (8.19%)	354 (4.80%)	

^1^ Chi-Square *p*-value; BOTH = combined PEG-/NEA-asparaginase.

**Table 6:** Multivariable analysis (MVA).

Effect	*p*-Value	OR (95% CI)
Study (Adults vs. Pediatric)	<0.0001	3.041 (2.589, 3.572)
ASP (BOTH vs. PEG)	<0.0001	1.359 (1.153, 1.601)
ASP (NEA vs. PEG)		1.771 (1.524, 2.058)

Footnote: MVA = multivariable analysis effect estimate reported directly by the original study authors. Adjusted ORs shown were obtained from author-generated logistic regression models using aggregated event counts.

**Table 7:** Association between B-cell/T-cell and adults vs. pediatrics on VTE.

		Study		
Var		Adults	Pediatric	*p*-Value
B-cell		(*N* = 1783)	(*N* = 8198)	
	Outcome, *n* (%)			<0.0001 ^1^
	Non-VTE	1728 (96.92%)	7745 (94.47%)	
	VTE	55 (3.08%)	453 (5.53%)	
T-cell		(*N* = 380)	(*N* = 1276)	
	Outcome, *n* (%)			0.0058 ^1^
	Non-VTE	353 (92.89%)	1121 (87.85%)	
	VTE	27 (7.11%)	155 (12.15%)	

^1^ Chi-Square *p*-value; OR = 1.838 (95% CI 1.382–2.443) for B-cell and 1.808 (95% CI 1.181–2.769) for T-cell.

**Table 8:** Multivariable analysis (MVA) of patient populations and immunophenotype.

Effect	*p*-Value	OR (95% CI)
Study (Pediatric vs. Adults)	<0.0001	1.828 (1.443, 2.316)
Cell (T-cell vs. B-cell)	<0.0001	2.370 (1.982, 2.833)

Footnote: MVA = multivariable analysis effect estimate reported directly by the original study authors. Adjusted ORs shown were obtained from author-generated logistic regression models using aggregated event counts.

**Table 9:** Association between B-cell/T-cell and development of VTE.

	Type of Cell		
	B-Cell (*N* = 11,539)	T-Cell (*N* = 1898)	*p*-Value
Outcome, *n* (%)			<0.0001 ^1^
Non-VTE	10,934 (94.76%)	1681 (88.57%)	
VTE	605 (5.24%)	217 (11.43%)	

^1^ Chi-Square *p*-value; OR = 2.333 (95% CI 1.981–2.747).

**Table 10:** Association between study type and development of VTE by gender.

		Gender		
Var		Adult	Pediatric	*p*-Value
Female		(*N* = 1195)	(*N* = 4002)	
	Outcome, *n* (%)			0.1719 ^1^
	Non-VTE	1150 (96.23%)	3814 (95.30%)	
	VTE	45 (3.77%)	188 (4.70%)	
Male		(*N* = 1532)	(*N* = 5160)	
	Outcome, *n* (%)			0.3973 ^1^
	Non-VTE	1446 (94.39%)	4840 (93.80%)	
	VTE	86 (5.61%)	320 (6.20%)	

^1^ Chi-Square *p*-value; OR = 1.260 (95% CI 0.904–1.755) for female and 1.112 (95% CI 0.870–1.421) for male.

**Table 11:** Association between adults and pediatrics and thrombosis rates by phase of treatment.

		Type of Study		
Phase		Adults	Pediatric	*p*-Value
Induction/re-induction		(*N* = 525)	(*N* = 12,689)	
	Outcome, *n* (%)			<0.0001 ^1^
	Non-VTE	487 (92.76%)	12,401 (97.73%)	
	VTE	38 (7.24%)	288 (2.27%)	
Intensification/consolidation /maintenance		(*N* = 0)	(*N* = 372)	
	Outcome, *n* (%)			
	Non-VTE	0 (0%)	356 (95.70%)	
	VTE	0 (0%)	16 (4.30%)	

^1^ Chi-Square *p*-value.

**Table 12:** Association between phases of treatment and VTE rates.

	Phases of Treatment		
	Induction/Re-Induction (*N* = 15,841)	Intensification/Consolidation/Maintenance (*N* = 2872)	*p*-Value
Outcome, *n* (%)			0.0773 ^1^
Non-VTE	15,445 (97.50%)	2816 (98.05%)	
VTE	396 (2.50%)	56 (1.95%)	

^1^ Chi-Square *p*-value; OR = 0.776 (95% CI (0.585, 1.029)).

**Table 13:** Association between steroids us and VTE rates.

	Steroid		
	Dex (*N* = 3247)	Pred (*N* = 4181)	*p*-Value
Outcome, *n* (%)			0.2013 ^1^
Non-VTE	3168 (97.57%)	4059 (97.08%)	
VTE	79 (2.43%)	122 (2.92%)	

^1^ Chi-Square *p*-value; OR = 0.830 (95% CI (0.623, 1.105)).

**Table 14:** Association between presence and absence of mediastinal mass and VTE rates.

	Mediastinal Mass		
	No (*N* = 3213)	Yes (*N* = 287)	*p*-Value
Outcome, *n* (%)			<0.0001 ^1^
Non-VTE	3019 (93.96%)	235 (81.88%)	
VTE	194 (6.04%)	52 (18.12%)	

^1^ Chi-Square *p*-value; OR = 3.444 (95% CI (2.467, 4.807)).

**Table 15:** Association between blood group and VTE rates.

	Blood Group		
	Non-O (*N* = 1216)	O (*N* = 907)	*p*-Value
Outcome, *n* (%)			0.0004 ^1^
Non-VTE	1086 (89.31%)	850 (93.72%)	
VTE	130 (10.69%)	57 (6.28%)	

^1^ Chi-Square *p*-value; OR = 0.560 (95% CI (0.405, 0.775)).

**Table 16:** Association between weight status and VTE rates.

	Weight Status		
	Normal/Underwt (*N* = 4791)	Overwt/Obese (*N* = 669)	*p*-Value
Outcome, *n* (%)			<0.0001 ^1^
Non-VTE	4459 (93.07%)	542 (81.02%)	
VTE	332 (6.93%)	127 (18.98%)	

^1^ Chi-Square *p*-value; OR = 3.147 (95% CI (2.518, 3.934)).

**Table 17:** Age groups. Association between type of study and development of VTE stratified by age grouping.

		Study		
Var		Mixed	Pediatric	*p*-Value
Age < 10		(*N* = 0)	(*N* = 14,357)	
	Outcome, *n* (%)			
	Non-VTE	0 (0%)	13,883 (96.70%)	
	VTE	0 (0%)	474 (3.30%)	
Age ≥ 10		(*N* = 1132)	(*N* = 2370)	
	Outcome, *n* (%)			0.7674 ^1^
	Non-VTE	1364 (90.15%)	2777 (90.43%)	
	VTE	149 (9.85%)	294 (9.57%)	

^1^ Chi-Square *p*-value; OR = 0.969 (95% CI (0.788, 1.193)) for age ≥ 10.

## Data Availability

No new data were created or analyzed in this study. Data sharing is not applicable to this article.

## Supplementary Materials

The following supporting information can be downloaded at: https://www.mdpi.com/article/10.3390/curroncol33060368/s1, Table S1a: PRISMA 2020 Checklist; Table S1b: PRISMA 2020 for Abstracts checklistt; Table S2a: Table Supplementary; Table S2b: Full electronic search strategies (PubMed, Ovid MEDLINE, Embase, CENTRAL, Google Scholar). Searches were conducted between 1994 and 2026, with the final search run on [2 March 2026]. All strategies were limited to English-language human studies.
