# Supplementary material for: Thrombosis-Associated Risk Factors in Pediatrics and Adults Treated with Asparaginase-Containing Chemotherapy for ALL: A Systematic Review and Meta-Analysis
Source: Curr Oncol. 2026 Jun 18;33(6):368. doi: 10.3390/curroncol33060368 (PMC13297999; doi:10.3390/curroncol33060368)
Supplement: Supplementary file 1 [file curroncol-33-00368-s001.zip › Table S1.pdf]

Table S1a: PRISMA 2020 Checklist.

Section/topic	Item No	Checklist item	Reported on Page Number/Line Number	Reported on Section/Paragraph
**TITLE**				
Title	1	Identify the report as a systematic review.	p.1, lines 1–3	Title page → Article title (“…A
**ABSTRACT**				
Abstract	2	See the PRISMA 2020 for Abstracts checklist (Table S1b).	p.1–2, lines 28–52	Abstract (entire structured abstract)
**INTRODUCTION**				
Rationale	3	Describe the rationale for the review in the context of existing knowledge.	p.2–3, lines 54–91	Introduction → Paragraphs 1–4
Objectives	4	Provide an explicit statement of the objective(s) or question(s) the review addresses.	p.3, lines 89–93, 101-104	Introduction → Final paragraph before Methods
**METHODS**				
Eligibility criteria	5	Specify the inclusion and exclusion criteria for the review and how studies were grouped for the syntheses.	p.3–4, lines 116–128	Methods → 2.1 Eligibility Criteria
Information sources	6	Specify all databases, registers, websites, organisations, reference lists and other sources searched or consulted to identify studies. Specify the date when each source was last searched or consulted.	p.4–5, lines 169–176	Methods → 2.5 Search Strategy
Search strategy	7	Present the full search strategies for all databases, registers and websites, including any filters and limits used.	p.4–5, lines 169–176 + Supplement	Methods → 2.5 Search Strategy (“Full reproducible strategies in Supplement”)

Selection process	8	Specify the methods used to decide whether a study met the inclusion criteria of the review, including how many reviewers screened each record and each report retrieved, whether they worked independently, and if applicable, details of automation tools used in the process.	p.4, lines 142–156	Methods → 2.3 Study Selection and Duplicate Removal
Data collection process	9	Specify the methods used to collect data from reports, including how many reviewers collected data from each report, whether they worked independently, any processes for obtaining or confirming data from study investigators, and if applicable, details of automation tools used in the process.	p.4, lines 142–156	Methods → 2.3 Study Selection and Duplicate Removal ("Data extraction used a standardized form… independently verified…")
Data items	10a	List and define all outcomes for which data were sought. Specify whether all results that were compatible with each outcome domain in each study were sought (e.g. for all measures, time points, analyses), and if not, the methods used to decide which results to collect.	p.3–4, lines 129–141	Methods → 2.2 Study Population and Treatment
	10b	List and define all other variables for which data were sought (e.g. participant and intervention characteristics, funding sources). Describe any assumptions made about any missing or unclear information.	p.5, lines 178–187	Methods → 2.6 Data Handling and Identification of Risk Factors

Study risk of bias assessment	11	Specify the methods used to assess risk of bias in the included studies, including details of the tool(s) used, how many reviewers assessed each study and whether they worked independently, and if applicable, details of automation tools used in the process.	p.4, lines 159–167	Methods → 2.4 Risk of Bias Assessment
Effect measures	12	Specify for each outcome the effect measure(s) (e.g. risk ratio, mean difference) used in the synthesis or presentation of results.	p.5, lines 189–216	Methods → 2.7 Statistical Analysis ("Effect estimates were extracted as odds ratios…")
Synthesis methods	13a	Describe the processes used to decide which studies were eligible for each synthesis.	p.3–4, lines 116–128	Methods → 2.1 Eligibility Criteria
	13b	Describe any methods required to prepare the data for presentation or synthesis, such as handling of missing summary statistics, or data conversions.	p.5, lines 178–187	Methods → 2.6 Data Handling ("Risk factors were abstracted…")
	13c	Describe any methods used to tabulate or visually display results of individual studies and syntheses.	p.5, lines 189–230	Methods → 2.7 Statistical Analysis ("Forest plots were created…")
	13d	Describe any methods used to synthesize results and provide a rationale for the choice(s). If meta-analysis was performed, describe the model(s), method(s) to identify the presence and extent of statistical heterogeneity, and software package(s) used.	p.5, lines 194–216	Methods → 2.7 Statistical Analysis
	13e	Describe any methods used to explore possible causes of heterogeneity among study results.	p.5, lines 201–207	Methods → 2.7 Statistical Analysis ("Substantial heterogeneity was anticipated…")
	13f	Describe any sensitivity analyses conducted to assess robustness of the synthesized results.	p.5, lines 208–216	Methods → 2.7 Statistical Analysis ("Sensitivity analyses were not performed…")

Reporting bias assessment	14	Describe any methods used to assess risk of bias due to missing results in a synthesis (arising from reporting biases).	Not performed; stated implicitly	Methods → 2.7 Statistical Analysis (no reporting bias assessment described)
Certainty assessment	15	Describe any methods used to assess certainty (or confidence) in the body of evidence for an outcome.	Not performed; not described	No certainty assessment section present
**RESULTS**				
Study selection	16a	Describe the results of the search and selection process, from the number of records identified in the search to the number of studies included in the review, ideally using a flow diagram.	p.6, lines 232–236 + Figure 1 (p.6)	Results → Figure 1 + accompanying text
	16b	Cite studies that met many but not all inclusion criteria ('near-misses') and explain why they were excluded.	p.4, lines 151–156	Methods → 2.3 Study Selection ("The remaining 156 studies were excluded…")
Study characteristics	17	Cite each included study and present its characteristics.	Tables 1–7, p.7–15	Results → Tables 1–7
Risk of bias in studies	18	Present assessments of risk of bias for each included study.	p.4, lines 159–167	Methods → 2.4 Risk of Bias Assessment (ROB described but not tabulated in Results)
Results of individual studies	19	For all outcomes, present, for each study: (a) summary statistics for each group (where appropriate) and (b) an effect estimate and its precision (e.g. confidence/credible interval), ideally using structured tables or plots.	Tables 1–7, p.7–15	Results → Tables 1–7

Results of syntheses	20a	For each synthesis, briefly summarise the characteristics and risk of bias among contributing studies.	p.6–7, lines 234–257	Results → Opening paragraphs + Table 1
	20b	Present results of all statistical syntheses conducted. If meta-analysis was done, present for each the summary estimate and its precision (e.g. confidence/credible interval) and measures of statistical heterogeneity. If comparing groups, describe the direction of the effect.	Tables 2–7, p.7–15	Results → Tables 2–7 + forest plots
	20c	Present results of all investigations of possible causes of heterogeneity among study results.	Forest plots (e.g., Figure 3-5, p.10-14)	Results → Figures 3, 4, 5 (I^2 values reported)
	20d	Present results of all sensitivity analyses conducted to assess the robustness of the synthesized results.	Methods → 2.7 Statistical Analysis ("Sensitivity analyses were not performed…")	Methods → 2.7 Statistical Analysis ("Sensitivity analyses were not performed…")
Reporting biases	21	Present assessments of risk of bias due to missing results (arising from reporting biases) for each synthesis assessed.	Not assessed	No reporting bias assessment included in
Certainty of evidence	22	Present assessments of certainty (or confidence) in the body of evidence for each outcome assessed.	Not performed	No certainty assessment section present
**DISCUSSION**				
Discussion	23a	Provide a general interpretation of the results in the context of other evidence.	p.15–18, lines 391–540	Discussion → Opening paragraphs
	23b	Discuss any limitations of the evidence included in the review.	p.15, lines 540–585	Discussion → Limitations paragraph
	23c	Discuss any limitations of the review processes used.	p.4, lines 142–148	Methods → 2.3 Study Selection ("…this deviation is acknowledged as a methodological limitation.")

	23d	Discuss implications of the results for practice, policy, and future research.	p.20, lines 595-602	Discussion → Final paragraphs ("Thromboprophylaxis is advised…")
**OTHER INFORMATION**				
Registration and protocol	24a	Provide registration information for the review, including register name and registration number, or state that the review was not registered.	p.2, line 49, p.4. line 165	Abstract → Conclusions ("This review was not registered.") Materials and Methods
	24b	Indicate where the review protocol can be accessed, or state that a protocol was not prepared.	p.4, lines 165–167	Methods → 2.4 Risk of Bias Assessment ("…no protocol was prepared…")
	24c	Describe and explain any amendments to information provided at registration or in the protocol.	Not applicable	No protocol existed; no amendments possible
Support	25	Describe sources of financial or non-financial support for the review, and the role of the funders or sponsors in the review.	p.2, line 49, p.20. line 164	Abstract → Conclusions ("…received no external funding.")
Competing interests	26	Declare any competing interests of review authors.	p.20. line 618 (end of manuscript)	Competing Interests section ("The authors declare no conflicts of interest.")
Availability of data, code and other materials	27	Report which of the following are publicly available and where they can be found: template data collection forms; data extracted from included studies; data used for all analyses; analytic code; any other materials used in the review.	p.20. 615-616 (end of manuscript)	Data Availability Statement ("Data available upon request…")

Table S1b. PRISMA 2020 for Abstracts checklist.

Section/topic	Item No	Checklist item	Reported on Page Number/Line Number	Reported on Section/Paragraph
**TITLE**				
Title	1	Identify the report as a systematic review.	p.1, lines 28–31	Abstract → Opening sentence (“This systematic review and meta-analysis…”)
**BACKGROUND**				
Objectives	2	Provide an explicit statement of the main objective(s) or question(s) the review addresses.	p.1, lines 29–31	Abstract → Background (“…evaluated thrombosis incidence and risk factors…”)
**METHODS**				
Eligibility criteria	3	Specify the inclusion and exclusion criteria for the review.	p.1, lines 32–38	Abstract → Methods (“Eligible studies were observational cohorts or clinical trials…”)
Information sources	4	Specify the information sources (e.g. databases, registers) used to identify studies and the date when each was last searched.	p.1, lines 32–33	Abstract → Methods (“Searches included Ovid MEDLINE, Embase…”)

Updated on September 21, 2020

Risk of bias	5	Specify the methods used to assess risk of bias in the included studies.	p.1, lines 36–38	Abstract → Methods ("Risk of bias was assessed using ROBINS I and RoB 2.")
Synthesis of results	6	Specify the methods used to present and synthesize results.	p.1, lines 36–38	Abstract → Methods ("Random effects models were used…")
**RESULTS**				
Included studies	7	Give the total number of included studies and participants and summarise relevant characteristics of studies.	p.1, lines 32–33	Abstract → Methods ("58 met inclusion criteria, totaling 23,655 patients…")
Synthesis of results	8	Present results for main outcomes, preferably indicating the number of included studies and participants for each. If meta-analysis was done, report the summary estimate and confidence/credible interval. If comparing groups, indicate the direction of the effect (i.e. which group is favoured).	p.1–2, lines 39–46	Abstract → Results ("Adults had significantly higher thrombosis rates…")
**DISCUSSION**				
Limitations of evidence	9	Provide a brief summary of the limitations of the evidence included in the review (e.g. study risk of bias, inconsistency and imprecision).	p.2, lines 45–46	Abstract → Results ("Findings were limited by substantial heterogeneity…")
Interpretation	10	Provide a general interpretation of the results and important implications.	p.2, lines 47–49	Abstract → Conclusions ("Thrombosis risk was multifactorial…")
**OTHER**				

Funding	11	Specify the primary source of funding for the review.	p.2, line 49	Abstract → Conclusions ("…received no external funding.")
Registration	12	Provide the register name and registration number.	p.2, line 49	Abstract → Conclusions ("This review was not registered.")
