# Supplementary material for: Thrombosis-Associated Risk Factors in Pediatrics and Adults Treated with Asparaginase-Containing Chemotherapy for ALL: A Systematic Review and Meta-Analysis
Source: Curr Oncol. 2026 Jun 18;33(6):368. doi: 10.3390/curroncol33060368 (PMC13297999; doi:10.3390/curroncol33060368)
Supplement: Supplementary file 1 [file curroncol-33-00368-s001.zip › Table S2.pdf]

**Table S2a:** Table Supplementary.					
Before conducting this meta-analysis, we surveyed literature on thrombosis risk factors and their significance in adults and children with acute lymphoblastic leukemia (ALL). The survey aimed to provide current insights and assist in shaping the study question and objectives.					
Adults					
Ref.#, Authors (year)	Study design	Treatment protocols	Sample size (n)	Risk factors	Statistical analysis. UVA / MVA, p-values
50. Underwood B et sl. 2020	Single-institution retrospective	CALGB 10403	Total 44 VTE= (18), No VTE= (26)	*Male sex *personal Hx. of thrombosis	UVA: Age at Dx. HR 1.03. 0.97-1.1. Male sex, hazard ratio (HR) of 3.45 (95% CI: 1.01–11.86, p = 0.049), and Personal Hx. of thrombosis, HR of 3.50 (95% CI: 2.05–7.06, p <.01. Family Hx of thrombosis HR2.75(0.61-12.34). oral contraceptive HR1.15 (0.2-6.69). MVA (personal history): HR of 2.73 (95% CI: 1.40–5.33, p = .003
**51. Zuurbier SM**. et al. 2015	Retrospectively analyzed	Dutch-Belgian HOVON-37 ALL (HO37)	240 patients, CVT= 9 (3.75%)	* post cycle 1 induction Trx. IT MTX.	n/a
8. Orvain C. et al. 2020	Randomized trial	GRAALL-2005 study	784 patients, thrombosis=122 (16%)	*Older Age, *High BMI and *high platelet count at Dx.	MVA: Older Age (OR, 1.23; 95% CI, 1.03-1.48; P = .02), a high BMI (OR, 1.05; 95% CI, 1.00-1.10; P = .03), and a high platelet count at Dx (OR, 1.03; 95% CI, 1.00-1.05; P = .03) For CVT: high hemoglobin level at Dx (OR, 1.17; 95% CI, 1.03-1.33; P = .01
52 De Stefano V. et al 2005	follow-up observational cohort study	n/a	379 acute leukemia, 69 was ALL. 7 (10.1%) VTE at Dx and post Trx.	*L-ASP post Trx.+ FV Leiden.	n/a
53 **Couturier M-A.** et al 2015	Retrospective study	GRAALL2003 GRAALL2005 and LL03	708 patients, CNS thrombosis 20 (3.1%)	*Med. # of L-ASP infusions, *Med. # of IT, *AT and Heparin Prx.	Med. # of L-ASP infusions, p<0.001 Med. # of IT, p<0.001 AT Prx p=0.0002 and Heparin (LMWH/UFH) Prx. p=0.025
31 Hunault-Berger M. et al 2008	Retrospective study	GOELAL02 or T-LBL/ALL GOELAL02	Total 214 patients	Oral contraceptives	Oral contraceptives (OR 4.33; 95% CI 1.04-18.1; P=0.03)
59 **Liang** J.et al 2017	Retrospective study.	n/a	122 patients ≥14 years. 46 treated with PEG-ASP and 76 with NEA	PEG-ASP > E coli ASP potential risk factors	PEG-ASP vs. NEA groups: longer duration of coagulation dysfunction (9.80±5.51 vs. 6.80±4.21 days; P=0.002)

Authors: 50=**Underwood B.** et al. 2020, 51=**Zuurbier SM**. et al. 2015, 8=Orvain C. et al. 2020, 52=De Stefano V. et al 2005, 53=**Couturier M-A.** et al 2015, 31=Hunault-Berger M. et al 2008, 54=**Liang** J.et al 2017.<br>cerebral venous thrombosis (CVT), AT=antithrombin, Prx=prophylaxis, IT=intrathecal, Pred=prednisone, PEG-ASP=pegylated-asparaginase, NEA= Native E. coli asparaginase, Dx=diagnosis,					
Pediatrics					
46.<br>**Silverman**<br>LB.et al 2001	Randomized study, retrospective	Protocol 91-01	377 patients Older vs. Younger patients	*Older children (9–18 years)	(15% vs 2%; P= .01)
38.<br>Athale UH.et al. 2018	Prospective analytical cohort study	DFCIALL05-001trial	Total 131 patients	*Age,<br>*Non-O blood and *blasts	MVA: Age at Dx 1.13 (1.01,1.26) 0.026<br>Non-O 3.64 (1.06,12.51) 0.040<br>Blasts 6.66 (0.82,53.85) 0.075
**55**<br>**El-Khoury**<br>H. et al 2021	A retrospective chart review	St. Jude Total XV therapy with minor medi cations	A total of 229 patients, 24 (10.5%) patients developed CSVT during treatment.	*Disease, *Mediastinal mass, *Max. TG level and *Larger BSA	MVA: INT/HR disease (OR: 6.879, 95% CI: 1.23538.304, P = 0.028), *Mediastinal mass (OR: 23.949, 95% CI: 5.606102.316, P = 0.000), Max TG levels of > 615 mg/dL (OR: 3.732, 95% CI: 1.00713.836, P = 0.049) and larger BSA (OR: 6.234, 95% CI: 1.69422.921, P = 0.006)
56<br>**Farinasso** L. et al 2007	prospectively evaluated study	Associazione Italiana di Emato-Oncologia Pediatrica (AIEOP)ALL95	All patients(n=56)<br>Non-CVL-VT (n=15(27%))<br>CVL-VT(n=41(73%))	*Catheter size/body surface ratio ≥9.6	MVA:<br>3(20%) vs. 25(61%)7.6 (1.6–35.9) p=0.01.<br>P (two-sided Fisher's exact). Catheter size/body surface ratio <9.6y, OR1, ≥9.6, OR7.6 (1.6–35.9)
57<br>**Klaassen**<br>ILM.et al. 2019	Retrospective study	Dutch Childhood Oncology Group (DCOG) ALL-10 protocol	Fifty- nine of 778 patients developed VTE (7.6%), with cerebral venous sinus thrombosis (CVST) in 26 of 59 patients (44.1%).	*Age ≥ 7, *T-cell ALL and *Med. risk disease ALL.	Age dichotomous (≥7 vs <7 y) OR 3.41 (1.72- 6.75). MVA OR 2.31. Male vs. Female UVA OR1.06 (0.41-2.71),<br>T- vs. B ALL subtype UVA OR1.80 (0.43- 7.46) and MVA OR1.60 (0.44- 7.30) ALL Med. risk vs. SR, HR, or unknown, OR4.14 (1.10-15.61), MVA OR2.64 (0.66-10.55)<br>High baseline absolute number of blasts in PB (≥42.5 vs <42.5), UVA OR 1.05 (0.04- 3.04)
35<br>Mateos MK et al 2019	retrospective cohort study	ANZCCSG Study VII, ANZCHOG Study 8, AIEOP-BFM ALL 2009-Study 9, COG	Fifty-two VTEs (5.1% ± 0.7%) were identified in the cohort of 1021 patients.	*Age ≥10 y, *Mediastinal mass and *Weight	MVA: Age ≥ 10years 0.036 1.97(1.05–3.72). Mediastinal mass 0.017 2.89(1.21–6.95). Weight 95th centile at diagnosis 0.001 2.94(1.54–5.59).<br>Age (continuous) 0.016 OR 1.01(1.00–1.01) Older age

		A5971 and BFM-95			(≥10years vs <10 y) 0.005, OR 2.32(1.30–4.13), T-cell <0.001, OR3.29(1.72–6.30). High risk group 0.027, OR2.08(1.09–3.98). Mediastinal mass <0.001,OR 3.79(1.81–7.95). Weight 0.001 OR3.00(1.59–5.66), BMI 0.062, OR2.14(0.96–4.77), ABO blood group(non O vs O) 0.16,OR 1.51(0.85–1.70)
**58** **Santoro** N. et al. 2013	Prospective, case-control study on VTE	AIEOP-BFM ALL2000 protocol	2042 patients, 48(2.4%) had VTE	*Male<br>*Dex =pred	n/a
**9** **Tuckuviene** R. et al. 2015	Prospective evaluation of thrombosis	NOPHO-ALL 2008	1038 patients, thrombosis =63 (6.1%)	Age ≥15 y, thrombosis after induction. Residual disease ≥ 5% after day 29	MVA:15–17 y 4.2(2.1–8.6) p < 0.0001<br>post induction. 4.0(2.1–7.7) p < 0.0001 total thrombosis. Residual disease ≥ 5% after day 29 2.3(0.9–5.5) 0.07 2.3(1.0–5.2) p=0.047
41 Prasca S et al. 2018	retrospective cohort	Standard-Risk (SR-ALL: CCG1991, AALL0331, AALL0932), High-Risk B-ALL (HR ALL: modified CCG1961 [PEG-ASP during Induction], AALL08P1, AALL0232, AALL1131), or T-ALL (AALL0434)	total of 294 patients, 27 (9.2%) developed thrombosis	*Obesity. majority of VTE were associated with a CVC (20/27, 74%) post first dose of PEG-ASP in Induction phase (19/27, 70%). Obese pts start Induction had a symptomatic VTE during therapy (19%, 12/64). Induction remained the highest risk phase for obesity-associated first symptomatic VTE 9/12 (75%)	MVA: Obese, OR = 3.8, 95%CI 1.5–9.6; global p = 0.008), compared to normal weight.
46=**Silverman** LB.et al 2001, 38=Athale UH.et al. 2018, 55=**El-Khoury** H. et al.2021, 56=**Farinasso** L. et al 2007, 57=**Klaassen** ILM.et al. 2019, 35=Mateos,MK. et al. 2019, 58=**Santoro** N. et al. 2013, 9=**Tuckuviene** R. et al. 2015, 41=Prasca S et al. 2018. INT=intermediate, HR= high risk					
Children and Adults combined studies below					

11<br>Toft N. et al. 2015	Observational study	NOPHO)<br>ALL2008 protocol	1509 patients (ages 1-45 y)	Age 10-17 and 18-45 y	1-9 y, 36/973 (3.6%) 1.0 (1.0-1.0). 10-17y, 40/222 (15.3%), 5.0 (3.1-8.2). 18-45y, 37/175(17.5%), 6.0 (3.6-10.1). increasing age was significantly predictive of thrombosis with the highest OR for patients15–17yrs, (OR5.4(95%CI: (2.6;11.0), P<0.0001),
4<br>Rank CU.et al. 2018	Prospectively; (toxicity: VTE) Retrospectively; all other	NOPHO<br>ALL2008 protocol	1772 patients (ages 1-45 y)	*10.0 to 17.9 years and ages 18.0 - 45.9 y<br>*enlarged lymph node<br>*mediastinal mass	ages 10.0 to 17.9 years (HRa, 4.9; 95%CI,3.1-7.8; P<0001) and ages 18.0 to45.9years (HRa,6.06; 95% CI, 3.65-10.1; P <.0001) compared with children younger than 10.0 years.
11=Toft N. et al. 2015 4=Rank CU.et al. 2018					

Supplementary Table S2b. Full electronic search strategies (PubMed, Ovid MEDLINE, Embase, CENTRAL, Google Scholar). Searches were conducted between 1994 and 2026, with the final search run on [2 March 2026]. All strategies were limited to English-language human studies.

PubMed (MEDLINE via PubMed)

("acute lymphoblastic leukemia"[MeSH Terms] OR "ALL"[Title/Abstract] OR "acute lymphoblastic leukaemia"[Title/Abstract])

AND

("thrombosis"[MeSH Terms] OR "thromboembolism"[Title/Abstract] OR "venous thromboembolism"[Title/Abstract] OR "blood clot"[Title/Abstract] OR "deep vein thrombosis"[Title/Abstract] OR "pulmonary embolism"[Title/Abstract])

AND

("asparaginase"[MeSH Terms] OR "pegaspargase"[Title/Abstract] OR "E. coli asparaginase"[Title/Abstract] OR "Erwinia asparaginase"[Title/Abstract])

AND

("risk factors"[MeSH Terms] OR "predictors"[Title/Abstract])

Filters: English, 1994–2026

Ovid MEDLINE

1. exp Leukemia, Lymphoblastic, Acute/

2. (acute lymphoblastic leukemia or ALL).ti,ab.

3. exp Thrombosis/

4. (thrombosis or thromboembolism or VTE or DVT or PE or blood clot).ti,ab.

5. exp Asparaginase/

6. (asparaginase or pegaspargase or Erwinia or E coli asparaginase).ti,ab.

7. exp Risk Factors/

8. 1 OR 2

9. 3 OR 4

10. 5 OR 6

11. 7

12. 8 AND 9 AND 10 AND 11

13. Limit 12 to English language and humans (1994–2026)

Embase (Ovid)

1. 'acute lymphoblastic leukemia'/exp

2. 'thrombosis'/exp

3. 'asparaginase'/exp

4. 'risk factor'/exp

5. (ALL OR acute lymphoblastic leukemia).ti,ab.

6. (thrombosis OR thromboembolism OR VTE OR DVT OR PE).ti,ab.

7. (asparaginase OR pegaspargase OR erwinia).ti,ab.

8. 1 OR 5

9. 2 OR 6

10. 3 OR 7

11. 4

12. 8 AND 9 AND 10 AND 11

13. Limit 12 to English language, humans and 1994–2026

Cochrane CENTRAL

(acute lymphoblastic leukemia OR ALL) AND

(thrombosis OR thromboembolism OR VTE OR DVT OR pulmonary embolism) AND

(asparaginase OR pegaspargase OR Erwinia OR E coli) AND

(risk factors)

Google Scholar

Google Scholar does not support reproducible line-by-line Boolean strategies; therefore, we provide a narrative description consistent with PRISMA guidance. We screened the first 200 records sorted by relevance using combinations of the following terms: "thrombosis", "thromboembolism", "acute lymphoblastic leukemia", "asparaginase", "pegaspargase", "risk factors", "pediatrics", and "adults.
